# The impact of community older adult care services on the quality of life of older adults in China: the mediating role of social adaptation

**DOI:** 10.3389/fpubh.2025.1544575

**Published:** 2025-04-29

**Authors:** Yuanfeng Liu, Shilin Yang

**Affiliations:** School of Public Administration and Law, Hunan Agricultural University, Changsha, China

**Keywords:** community older adult care services, social adaptation, quality of life of older adults, Chinese older adults, mediating effect

## Abstract

**Background:**

With the deepening trend of aging in China, community older adult care services occupy an important position in China’s older adult care system. This paper aims to explore the impact of different types of community older adult care services on the quality of life of older adults and to analyze its underlying influencing mechanism.

**Methods:**

The research data are obtained from the 2020 China Longitudinal Aging Social Survey data. The Structural Equation Model is used to examine the relationship between of three types of community older adult care services on older adults’quality of life.

**Results:**

(1) Three types of community older adult care services are positively associated with quality of life of older adults. Compared to basic older adult care services, extended older adult care services have a more significant impact on older people’s quality of life. (2) It is found that the impacts of three types of community older adult care services on the quality of life of older adults are heterogeneous in terms of age, registered residence, and disablement status. (3) Social adaptation mediated the relationship between three types of community older adult care services and older adults’ quality of life.

**Discussion:**

The results of this study show that three types of community older adult care services could improve the quality of life of older adults, and social adaptation plays a mediating role between three types of community older adult care services and quality of life. Therefore, it is necessary to strengthen the supply of community older adult care services, especially extended older adult care services. By accurately identifying the needs of the older adults and optimizing service supply, giving the older adults more useful services, improving their attitudes towards society and helping them better adapt to society, thereby improving the quality of life of older adults. This study provides a basis for optimizing community older adult care services and improving the quality of life of the older adults, and emphasizes the key role of social adaptation, which has important practical guiding significance for promoting the sustainable development of the older adult care service system.

## 1 Introduction

According to data released by China’s National Bureau of Statistics, by 2022, there are about 280.04 million older adults over the age of 60, accounting for 19.8% of China’s total population. In the current context of rapid aging in China, community older adult care services occupy an important position in the older adult care service system and are an important measure to cope with the problem of older adult care. Community older adult care services not only needs to meet the basic needs of the older adults, but also assumes the function of promoting the social adaptation and maintaining the social connection of the older adults. In addition, community older adult care services significantly affect the quality of life of the older adults by providing them with various supports and services. We not only need to study the difference in the impact of different types of community older adult care service supply on the quality of life of the older adults population, but also need to pay attention to the internal logic of social adaptation between community older adult care services and the quality of life of the older adults. This will help optimize resource allocation, improve service quality, and promote the sustainable development of community older adult care service. It also has far-reaching implications for improving the overall quality of life of the older adults. Therefore, based on the current predicament faced by China, what is the relationship between community older adult care services and the quality of life of older adults? Which kind of community older adult care services can better improve their quality of life? Will community older adult care services impact the social adaptations of older adults? Can it focus on improving the quality of life of older adults by enhancing their social adaptability?

For this reason, based on the data of the 2020 China Longitudinal Aging Social Survey (CLASS), this paper examines the impact of different types of community older adult care services on the quality of life of older people, analyzes the heterogeneity of the effect of community older adult care services in different older groups, and explores the mediating mechanism of social adaptation between different categories of community older adult care services and the quality of life of older adults. It provides theoretical support and perspective for the research in related fields.

## 2 Literature review

### 2.1 Research on influencing factors of quality of life of the older adults

The quality of life of older adults has always been a hot topic in the academic world. Older adults’ quality of life is affected by many factors such as the individual, family, living environment, and so on. From the perspective of individuals, the self-esteem (1), educational level and financial status of the older adults (2) will have an impact on their quality of life. In addition, the behavior and lifestyle of the older adults also affect their quality of life (3). For example, physical activity helps to improve the quality of life of older people (4). Reducing smoking and drinking will improve their quality of life (5). Poor sleep quality is likely to have a negative impact on older adults (6).

From the perspective of family, The family, as an important informal support for older adults, is an important factor influencing their quality of life (7, 8). The quality of life of the older adults with normal family function is higher. In contrast, those with mild or severe family dysfunction had significantly lower quality of life (9).Studies have shown that older people who live only with their spouse have a better quality of life (10).Older people who are cared for by their children have a better quality of life (11). Caring for grandchildren also affects the quality of life of older people, with the older and more numerous the grandchildren, the lower the quality of life of older people (12).

From the perspective of social environment, the living environment of the older adults will affect their quality of life. Higher living environment quality will improve the satisfaction of the older adults to life (13, 14). The more age-friendly the community, the better the quality of life (15). There are also differences in quality of life among older people in different regions. Older people living in the suburbs have a lower quality of life than those living in urban areas (16). In this information age, the use of the Internet can improve the health of the older adults (17) and can help them keep in close touch with family and friends, which has a positive impact on their quality of life (18–20). The higher the use of information and communication equipment by older people, the more positive the evaluation of quality of life (21). In recent years, some scholars have also paid attention to The impact of community older adult care services on the quality of life of older adults.

### 2.2 Research on community older adult care services and quality of life of the older adults

Community older adult care services are defined as the organized provision of a series of diversified, specialized, and convenient older adult services to older adults by the community (22). The content mainly includes daily life care, spiritual and emotional support, health care services, etc. (23). Older adults’ quality of life is an important tool for assessing the effectiveness of community older adult care services (24). At present, scholars have two primary points of view about the research on quality of life and community older adult care services: positive and negative. Scholars holding the positive viewpoint believe that community older adult care services are beneficial to improving the quality of life of the older adults (25–27). By providing older adult services, the community further supports older adults in independent living (28), expand their social network (29), and improves their quality of life. However, the scholars who hold the negative viewpoint believe that the influence of community older adult care services on the older adults people’s quality of life is not obvious, and even negatively correlates with their quality of life (30). There are problems such as the mismatch between the supply and demand of community older adult care services, which is not conducive to improving their quality of life (31). Furthermore, community older adult care services have no impact on the quality of life because of the low level of professionalism and poor quality of the supply of these services (32).

Consequently, it can be found that most studies generally agree that community older adult care services have an impact on the quality of life of the older adults, but there is still disagreement on the direction of the impact, and there is still room for expansion in the research. In terms of research content, little literature has explored the relationship between different types of community older adult care services and the quality of life of older people. In terms of research perspectives, much of the literature often analyzes the use of community older adult care services, and few scholars have conducted research from the perspective of the supply of community older adult care services.

### 2.3 Research on community older adult care services, social adaptation and the quality of life of older adults

Social adaptation refers to the process of maintaining balance and coordination with environment, society and interpersonal communication through self-regulation when individuals face the changing natural environment and social environment (33). There is an influence relationship between community aged care service and social adaptation of the older adults (34). Studies have shown that social support is closely related to individual social adaptation, and the higher the level of social support, the better the social adaptation ability of the older adults (35). As an important form of social support, community older adult care services can provide relevant support to the older adults to help them adapt to the society (36, 37). Therefore, community older adult care services help enhance the social adaptability of older persons.

In addition, some scholars believe that there is a close relationship between social adaptation and quality of life. Research has shown that social adaptation is closely related to the quality of life (38). Social adaptation can improve the quality of life of older adults (39). After entering the old age, the older adults can actively adjust their attitude, concept and mentality to face the changes of physical, mental and social environment, which helps to improve the quality of life of the older adults (40). It can be seen that social adaptation have an impact on older adults’ quality of life.

Based on these, there are the impact of community older adult care services on social adaptation and the impact of social adaptation on quality of life. We hypothesized that social adaptation can possibly mediate the link between community older adult care services and quality of life of older adults.

Hence, this paper will use structural equation model to explore the impact direction of three categories of community older adult care services, including total community older adult care services, basic community older adult care services, and extent community older adult care services, on the quality of life of older adults, and to reveal the intrinsic mechanism of social adaptation in the impact of these three types of community older adult care services on their quality of life.

## 3 Materials and methods

### 3.1 Data and research sample

This study uses data from the 2020 China Longitudinal Aging Social Survey, which is a large-scale nationwide, continuous survey implemented by the Survey and Data Center of Renmin University of China. The first nationwide baseline survey was conducted in 2014 and has been followed up every 2 years since then. The purpose of the survey is to gain a comprehensive understanding of the living conditions of the older adults in China and to provide a data basis for the formulation and improvement of policies on aging. The survey utilized a stratified multi-stage probability sampling method, selecting county-level areas (including counties, county-level cities, and districts) as the primary sampling unit and villages/habitat committees as the secondary sampling unit, covering a total of 476 villages/habitat committees in 30 provinces/autonomous regions/municipalities directly under the central government across the country, and succeeded in obtaining a sample of 11,398 persons aged 60 and above. The sampling design and implementation of the project ensured that the data were well-represented nationwide. According to the research situation in this paper, the data are processed, and the older adult samples with cognitive impairment are deleted (N = 467). At the same time, in order to ensure the reliability of data analysis and research results, the samples of older adults who chose “do not know” in the explanatory variables questionnaire are deleted (N = 5,897). Because the questionnaire had the option of being “unable to answer,” 382 samples lacking key variables and control variables are excluded. 4,652 valid samples are obtained after removing samples of extreme values of core variables and control variables.

### 3.2 Variable setting

#### 3.2.1 Dependent variable

The dependent variable is the quality of life of older adults. Combined with related studies, self-assessed health, depression status, and life satisfaction are used as measures of the latent variable “quality of life” in this study. Self-assessed health is a comprehensive evaluation index reflecting the physical health of the older adults (41), using the questionnaire “How do you feel about your current physical health?” to measure the self-assessed health. There are five options from “very healthy” to “very unhealthy.” By reversing the answer options, the higher the score, the better the health condition. Depression status is measured according to the CES-D scale in the CLASS questionnaire, the coefficient of confidence is 0.689. The questionnaire contained nine items describing the psychological condition of the respondents in the past week, including “Did you find yourself in a good mood this past week?,” “Have you felt lonely in the past week?,” “Have you felt sad in the past week?,” “Have you had a good week this past week?,” “Have you felt like eating for the past week?,” “Have you had trouble sleeping in the past week?,” “Have you felt useless in the past week?,” “Did you feel like you had nothing to do in the past week?,” “Have you had a lot of fun in your life this past week?.” The answer options are “no”, “sometimes”, and “often”, with 1 being “no” and 3 being “often.” Of the nine items, there are three positive and six negative items, for which the six negative items are reverse coded. The higher the score, the lower the level of depression. Simultaneously calculate the mean of the answers to 9 questions as a score for the older adults people’s depression status. Life satisfaction is determined by the question “Overall, are you satisfied with your current life?.” The questionnaire has five response options ranging from “very satisfied” to “very dissatisfied,” which are reversed. “Very satisfied” is assigned a value of 5 and “very dissatisfied” is assigned a value of 1. The higher the value, the higher the level of satisfaction.

#### 3.2.2 Independent variable

The independent variable of this study is community older adult care services. We explore The impact of community older adult care services on the quality of life of older adults at the overall level and different types of levels, respectively. According to the questionnaire, “Does your community provide the following services?.” The answer includes nine options: home visits, older adult service hotline, accompanying to see the doctor, help with daily shopping, legal aid, housekeeping, older adult dining tables or meal delivery, day-care centers or homes for the older adult, and psychological counseling. The answer to each service option was “yes,” “no” and “do not know.” To eliminate interference, the samples with “do not know” answer options for all nine services are deleted. Answering “yes” takes the value of 1, while answering “no” takes the value of 0. These nine options are used to measure variable “total community older adult care services.”

In addition, we further explore the impact of different types of community older adult care services on the quality of life of the older adults. Community older adult care services are categorized into basic community older adult care services and extended community older adult care services based on Maslow’s theory of needs. Maslow’s theory of needs divides human needs into five levels from low to high: physiological needs, safety needs, emotional and belonging needs, respect needs, and self-actualization needs. Basic services correspond mainly to the physiological and safety needs in Maslow’s theory of needs. In the nine services above, the five services, including accompanying to see the doctor, helping with daily shopping, housekeeping, older adult dining tables or meal delivery, and day-care centers or homes for the older adult, are the services that maintain the basic life of older adults. They are used to measure the variable “basic community older adult care services.” Extended services correspond to the higher level needs in Maslow’s theory of needs, that is, emotional and belonging needs, respect needs, and self-actualization needs. The four services, including home visiting, older adult service hotlines, legal aid, and psychological counseling, are referred to as “extended services,” which are used to measure the variable “extended community older adult care services.” For the definition and categorization of community older adult care services indicators, see Table 1.

**Table 1:** Definition and categorization of community older adult care services indicators.

	Indicators	Definition of indicators
Overall level	Total community older adult care services	Home visiting; Older adult service hotline; Accompanying to see the doctor; Help with daily shopping; Legal aid; Housekeeping; Older adult dining tables or meal delivery; Day-care centers or homes for the older adult; Psychological counseling
Different types	Basic community older adult care services	Accompanying to see the doctor; Helping with daily shopping; Housekeeping; Older adult dining tables or meal delivery; Day-care centers or homes for the older adult
	Extended community older adult care services	Home visiting service; Older adult service hotline; Legal aid; Psychological counseling

#### 3.2.3 Mediating variable

The mediating variable is the social adaptation of older adults. In this study, the variable of older adults’ social adaptation is measured using the social adaptation scale, which is sourced from Section E of the CLASS questionnaire, and the coefficient of confidence is 0.821. The answer has five levels of “full compliance,” “relatively compliance,” “general,” “relatively non-compliance” and “complete non-compliance.” The questionnaire included eight questions, such as “If the opportunity arises, I would be happy to participate in some of the work of village/neighborhood committees,” “I often want to do something for society again,” “I like studying now,” “I think I’m still a useful member of society,” “Society changes so fast that it is difficult for me to adapt to the change,” “Now, more and more opinions make me difficult to accept,” “Nowadays more and more new social policies make me difficult to accept,” “Social changes are becoming more and more detrimental to the older adult.” The first four questions are positive questions, with higher scores indicating greater adaptability. The last four questions are negative questions, which are reverse questions, meaning that the higher the score, the less adaptive. Each option is adjusted to score positively. Then the scores of each question item are summed up as the scores of the social adaptation of older adults. The higher the score, the stronger the social adaptability of older adults, conversely, the weaker the social adaptability.

#### 3.2.4 Control variables

The control variables in this paper contain gender, age, marital status, education level, living status, number of living children, intensity of social support, chronic diseases, basic mobility, daily mobility, the presence of pension insurance, and personal income, respectively. In particular, the personal income variable was logarithmic and intended to eliminate the effect of heteroskedasticity.

### 3.3 Models and methods

In order to explore the mechanism of the impact of various types of community older adult care services on the quality of life of older adults, we use structural equation modeling for estimation. Structural equation modeling contains measurement model and structural model. The measurement model is used to reflect the relationship between latent variables and their explicit variables, and its mathematical expression is shown below:


(1) $$Y=\Lambda_{y}\eta +\varepsilon$$

The structural equation is:


(2) η=βα+Γξ+τ

Equation 1, Y is a set of indicator variables for the quality of life of older adults, with three indicators for self-assessed health, depression status, and life satisfaction, respectively. η denotes the endogenous latent variable quality of life. $\Lambda_{y}$ is the factor loading matrix of Y on η . ε is the residual term of the equation. Equation 2, α and ξ represent the variables influencing endogenous latent variables. β and Γ respectively denote the impact relationships of α and ξ on the endogenous latent variables, and τ is a perturbation term in the structural model.

## 4 Results

### 4.1 Participant characteristics

The descriptive results of the variables are shown in Table 2. The average age of older adults was 71 years old, the minimum age was 60 years old, the maximum age was 97 years old, and the proportion of men and women was relatively balanced. More than half of older adults had a medium level of education. 74.79% had a spouse. Fewer older persons live alone. The number of living children was concentrated in the range of 1–3. The intensity of social support for older adults is in the lower middle range. More than 80% of older adults suffer from chronic diseases. A higher percentage of older adults with ADLs and BADLs are in better condition. The personal income of most of the older adults people interviewed is at a low level. The proportion receiving pensions is relatively large.

**Table 2:** Descriptive statistics of variables.

	Variables	Variable interpretation	Mean	Std	Min	Max
Dependent variable	Quality of life	Self-assessed health	3.368	0.879	1	5
		Depression status	2.211	0.371	1	3
		Life satisfaction	3.763	0.857	1	5
Independent variable	Total community older adult care services	No = 0; Yes = 1	0.390	0.488	0	1
	Basic community older adult care services	No = 0; Yes = 1	0.222	0.416	0	1
	Extended community older adult care services	No = 0; Yes = 1	0.368	0.482	0	1
Mediating variable	Social adaptation	Continuous variable	23.83	3.928	8	40
Control variables	Gender	Female = 0; male = 1	0.503	0.500	0	1
	Age	Actual age of respondents	71.64	6.493	60	97
	Marriage	Widowed, divorced,unmarried = 0; married = 1	0.748	0.434	0	1
	Education	Illiteracy = 0; primary school, junior high school = 1; senior high school and above = 2	0.857	0.566	0	2
	Living status	Living with others = 0; Live alone = 1	0.101	0.301	0	1
	Number of children alive	The sum of the number of sons and daughters alive	2.361	1.306	0	8
	Social support intensity	The score for obtaining help and support from friends and family, with higher scores indicating greater intensity of support.	4.603	1.918	0	10
	Chronic disease	Do not have a chronic illness = 0; Have a chronic illness = 1	0.821	0.383	0	1
	ADL	Assessment of Activities of Daily Living	24.14	2.271	9	25
	BADL	Assessment of Basic Activities of Daily Living	32.48	1.747	11	33
	Pension	Without enjoying pension = 0; enjoy pension = 1	0.797	0.402	0	1
	Personal income	Take the logarithm of an individual’s total annual income	3.767	4.549	0	16.12

### 4.2 Baseline regression results

Table 3 displays the results of structural equation estimation of different types of community older adult care services on the quality of life of older adults.

**Table 3:** Results of structural equation estimation of community older adult care services on the quality of life of older adults.

Model	Variables	(1)	(2)	(3)
Structural model	Dependent variable: Quality of life			
	Independent variable			
	Total community older adult care services	0.291*** (0.021)		
	Basic community older adult care services		0.056** (0.027)	
	Extended community older adult care services			0.303*** (0.021)
	Control variable			
	Gender	0.059*** (0.020)	0.062*** (0.021)	0.059*** (0.020)
	Age	−0.008*** (0.002)	−0.008*** (0.002)	−0.008*** (0.002)
	Marriage	0.036 (0.028)	0.046 (0.030)	0.036 (0.028)
	Education	0.086*** (0.019)	0.108*** (0.020)	0.089*** (0.018)
	Living status	−0.140*** (0.039)	−0.123*** (0.041)	−0.141*** (0.038)
	Number of children alive	0.006 (0.008)	0.002 (0.008)	0.004 (0.008)
	Social support intensity	−0.001 (0.005)	−0.001 (0.005)	−0.001 (0.005)
	Chronic disease	−0.360*** (0.028)	−0.333*** (0.029)	−0.357*** (0.028)
	ADL	0.039*** (0.007)	0.042*** (0.007)	0.038*** (0.007)
	BADL	0.069*** (0.009)	0.069*** (0.009)	0.069*** (0.009)
	Pension	−0.039 (0.027)	−0.019 (0.028)	−0.038 (0.026)
	Personal income	0.007*** (0.003)	0.011*** (0.003)	0.008*** (0.002)
Measurement model	Self-assessed health	1^a^	1^a^	1^a^
	Depression status	0.355*** (0.016)	0.327*** (0.016)	0.358*** (0.016)
	Life satisfaction	0.583*** (0.038)	0.547*** (0.040)	0.582*** (0.038)
Fitting index	N	4,652	4,652	4,652
	R2(CD)	0.243	0.192	0.236
	CFI	0.921	0.903	0.918
	RMSEA	0.042	0.042	0.040
	SRMR	0.018	0.019	0.018

The factor load of “a” used to mark the first measurement index is set to 1 by default by SEM. *p < 0.1, **p < 0.05, ***p < 0.01. Numbers in parentheses are robustness standard errors.

In the structural model, model (1) presents the results of total community older adult care services on the quality of life. The results show that total community older adult care services have a significant positive impact on the quality of life of older adults ( β = 0.291, *p* < 0.01). From the regression results of model (2), it can be seen that basic community older adult care services are significantly correlated with the quality of life of older adults, and the coefficient of the path of influence is positive ( β = 0.056, *p* < 0.05). Models (3) show that extended community older adult care services ( β = 0.303, *p* < 0.01) are positively and significantly correlated with quality of life. These results all indicate that community older adult care services have a contributory effect on the quality of life of older adults. In the measurement model section, the measures of self-assessed health, depression status, and life satisfaction on the quality of life of older adults are significantly positive at the 1% level, which suggests that all of the measured variables of this latent variable are measured at a high level.

In addition, this table reports the fit metrics of the three models.The tests of the great likelihood ratios are all significant at the 1% level, and the RMSEA and SRMR are all less than 0.05, and the CFI metrics are all greater than 0.9. These metrics indicate that all three structural equation models are relatively well-fitted.

### 4.3 Robustness test

#### 4.3.1 Addition of more control variables

To minimize the impact of omitted variables on the results, we add more control variables to the original model. The control variables added include political status (Non-party members = 0; Party membership = 1), registered residence (rural = 0; urban = 1), religious affiliation (no affiliation = 0; affiliation = 1), and respondents’ smoking status (non-smoking = 0; smoking = 1). The results are shown in Table 4.

**Table 4:** Robustness test results for adding more control variables.

Model	Variables	(1)	(2)	(3)
Structural model	Dependent variable: Quality of life			
	Independent variable			
	Total community older adult care services	0.287*** (0.021)		
	Basic community older adult care services		0.052* (0.028)	
	Extended community older adult care services			0.298*** (0.021)
	Control variable			
	Gender	0.027 (0.023)	0.032 (0.024)	0.029 (0.023)
	Age	−0.008*** (0.002)	−0.008*** (0.002)	−0.008*** (0.002)
	Marriage	0.037 (0.028)	0.046 (0.030)	0.036 (0.028)
	Education	0.083*** (0.019)	0.099*** (0.020)	0.085*** (0.019)
	Living status	−0.138*** (0.038)	−0.121*** (0.040)	−0.139*** (0.038)
	Number of children alive	0.005 (0.008)	0.003 (0.008)	0.004 (0.008)
	Social support intensity	−0.001 (0.005)	−0.001 (0.005)	−0.001 (0.005)
	Chronic disease	−0.357*** (0.028)	−0.332*** (0.029)	−0.354*** (0.028)
	ADL	0.038*** (0.007)	0.041*** (0.007)	0.038*** (0.007)
	BADL	0.068*** (0.009)	0.067*** (0.009)	0.068*** (0.009)
	Pension	−0.040 (0.027)	−0.019 (0.028)	−0.038 (0.026)
	Personal income	0.007*** (0.003)	0.010*** (0.003)	0.007*** (0.003)
	Political status	0.100* (0.052)	0.095* (0.055)	0.102** (0.052)
	Registered residence	0.000 (0.022)	0.031 (0.023)	0.002 (0.022)
	Religious affiliation	0.196*** (0.051)	0.023*** (0.053)	0.191*** (0.051)
	Smoking status	0.090*** (0.027)	0.093*** (0.028)	0.084*** (0.026)
Measurement model	Self-assessed health	1^a^	1^a^	1^a^
	Depression status	0.356*** (0.016)	0.329*** (0.016)	0.359*** (0.016)
	Life satisfaction	0.586*** (0.037)	0.555*** (0.039)	0.584*** (0.037)
Fitting index	N	4,652	4,652	4,652
	R2(CD)	0.242	0.202	0.243
	CFI	0.920	0.901	0.917
	RMSEA	0.035	0.037	0.035
	SRMR	0.015	0.016	0.015

The factor load of “a” used to mark the first measurement index is set to 1 by default by SEM. *p < 0.1, **p < 0.05, ***p < 0.01. Numbers in parentheses are robustness standard errors.

According to the results in Table 4, the regression coefficients of total community older adult care services ( β = 0.287, *p* < 0.01) on the quality of life remain significant at the 1% level. Basic older adult care services ( β = 0.052, *p* < 0.1) and extended older adult care services ( β = 0.298, *p* < 0.01) also remain significant and positive on quality of life. It can be seen that the significance, as well as the coefficients, remain consistent with the above regression results after adding control variables, indicating that the above estimations are robust.

#### 4.3.2 Change in estimation method

To test the robustness of the regression results, we test this using two new estimation methods. One is the great likelihood estimation plus robust standard errors (ml + robust). The other is the estimation for 1,000 repeated samples (bootstrap). The results are shown in Table 5.

**Table 5:** Robustness test results of the transformation estimation method.

Model	Variables	(1)	(2)	(3)	(4)	(5)	(6)
		Ml + robust	Bootstrap	Ml + robust	Bootstrap	Ml + robust	Bootstrap
Structural model	Dependent variable: Quality of life						
	Independent variable						
	Total community older adult care services	0.291*** (0.021)	0.291*** (0.021)				
	Basic community older adult care services			0.056** (0.028)	0.056** (0.027)		
	Extended community older adult care services					0.303*** (0.021)	0.303*** (0.021)
	Control variable						
	Gender	0.059*** (0.020)	0.059*** (0.020)	0.062*** (0.021)	0.062*** (0.021)	0.059 (0.020)	0.059 (0.020)
	Age	−0.008*** (0.002)	−0.008*** (0.002)	−0.008*** (0.002)	−0.008*** (0.002)	−0.008*** (0.002)	−0.008*** (0.002)
	Marriage	0.036 (0.029)	0.036 (0.029)	0.046 (0.030)	0.046 (0.029)	0.036 (0.028)	0.036 (0.029)
	Education	0.086*** (0.019)	0.086*** (0.020)	0.108*** (0.020)	0.108*** (0.020)	0.089*** (0.019)	0.089*** (0.020)
	Living status	−0.140*** (0.038)	−0.140*** (0.038)	−0.123*** (0.041)	−0.123*** (0.040)	−0.141*** (0.038)	−0.141*** (0.038)
	Number of children alive	0.006 (0.008)	0.006 (0.008)	0.002 (0.008)	0.002 (0.008)	0.004 (0.008)	0.004 (0.008)
	Social support intensity	−0.001 (0.005)	−0.001 (0.005)	−0.001 (0.005)	−0.001 (0.006)	−0.001 (0.005)	−0.001 (0.005)
	Chronic disease	−0.360*** (0.029)	−0.360*** (0.029)	−0.333*** (0.030)	−0.333*** (0.029)	−0.357***(0.029)	−0.357*** (0.029)
	ADL	0.039*** (0.007)	0.039*** (0.007)	0.042*** (0.008)	0.042*** (0.008)	0.038*** (0.007)	0.038*** (0.007)
	BADL	0.069*** (0.009)	0.069*** (0.009)	0.069***(0.010)	0.069*** (0.010)	0.069*** (0.009)	0.069*** (0.009)
	Pension	−0.039 (0.027)	−0.039 (0.028)	−0.019 (0.028)	−0.019 (0.028)	−0.038 (0.027)	−0.038 (0.028)
	Personal income	0.007*** (0.003)	0.007*** (0.003)	0.011*** (0.003)	0.011*** (0.003)	0.007*** (0.003)	0.007*** (0.003)
Measurement model	Self-assessed health	1^a^	1^a^	1^a^	1^a^	1^a^	1^a^
	Depression status	0.355*** (0.017)	0.355*** (0.018)	0.327*** (0.018)	0.327*** (0.018)	0.358*** (0.018)	0.358*** (0.018)
	Life satisfaction	0.583*** (0.043)	0.583*** (0.044)	0.547*** (0.047)	0.547*** (0.046)	0.582*** (0.042)	0.582*** (0.044)
Fitting index	N	4,652	4,652	4,652	4,652	4,652	4,652
	R2(CD)	0.235	0.235	0.192	0.192	0.236	0.236
	CFI		0.921		0.903		0.918
	RMSEA		0.039		0.042		0.040
	SRMR	0.018	0.018	0.019	0.019	0.018	0.018

The factor load of “a” used to mark the first measurement index is set to 1 by default by SEM. *p < 0.1, **p < 0.05, ***p < 0.01. Numbers in parentheses are robustness standard errors.

According to the results shown in Table 5, the effect of total community older adult care services on the quality of life of older adults remains significantly positive at the 1% level after using the new estimation method( β = 0.291, *p* < 0.01) ( β = 0.291, *p* < 0.01). Basic older adult care services have a significant contribution to the quality of life ( β = 0.056, *p* < 0.05)( β =0.056, p < 0.05). Extended older adult care services also remain consistent with the previous section in terms of significance and direction of regression ( β = 0.303, *p* < 0.01) ( β = 0.303, *p* < 0.01). These results suggest that total community older adult care services, basic community older adult care services, and extended community older adult care services have positive contributions to the quality of life of older persons. The results confirm that the above regression results are robust.

### 4.4 Group difference analysis

The above study of the relationship between community older adult care services and older adults’ quality of life is conducted from the perspective of the full sample. However, considering the differences between groups, we conduct grouping tests on the entire sample from three dimensions. It aims to study the impact of three types of community older adult care services on the quality of life of different older adult groups. Table 6 presents the results.

**Table 6:** Heterogeneity analysis results.

Variables	Age		Registered residence		Disablement status	
	(1)	(2)	(3)	(4)	(5)	(6)
	Younger old adults	Older to oldest old adults	Urban	Rural	Disabled	Non-disabled
Total community older adult care services	0.323*** (0.032)	0.256*** (0.028)	0.213*** (0.030)	0.379*** (0.032)	0.219** (0.086)	0.295*** (0.022)
Basic community older adult care services	0.113*** (0.038)	−0.012 (0.038)	−0.017 (0.034)	0.168*** (0.049)	−0.118 (0.113)	0.065** (0.028)
Extended community older adult care services	0.320*** (0.031)	0.278*** (0.028)	0.229*** (0.029)	0.387*** (0.032)	0.193** (0.087)	0.309*** (0.022)
Control variable	yes	yes	yes	yes	yes	yes
*N*	2,003	2,649	2,245	2,407	300	4,352

*p < 0.1, **p < 0.05, ***p < 0.01. Numbers in parentheses are robustness standard errors.

#### 4.4.1 Age group differences

Due to the differences in physical functions, psychological states, and lifestyles among older adult groups of different ages, their acceptance and effectiveness of community older adult care services may also vary. Therefore, exploring the effects of community older adult care services on the quality of life of older adult groups of different ages will help to continuously improve the services. According to the relevant literature (42), we classify older adults aged 60–69 as younger old adults, and older adults aged 70 and above as older to oldest old adults. The results in Table 6 show that total community older adult care services ( β = 0.323, *p* < 0.01), basic older adult care services ( β = 0.113, *p* < 0.01), and extended older adult care services ( β =0.320, *p* < 0.01) all significantly improve the quality of life of younger old adults. For older to oldest old adults, community older adult care services ( β = 0.256, *p* < 0.01) and extended older adult care services ( β = 0.278, *p* < 0.01) had a significant contribution to their quality of life, while basic older adult care services ( β = − 0.012, *p* > 0.1) had no significant effect on quality of life.

#### 4.4.2 Registered residence group differences

Classifying the older adult population in urban and rural areas not only helps to better understand their needs and service status but also provides an important basis for policy-making and implementation. We classify older adults based on their registered residence, dividing them into urban and rural. It can be found that the effect of community older adult care services on the quality of life of both urban and rural older adults is significant. Total community older adult care services positively affect the quality of life of both urban older adults ( β = 0.213, *p* < 0.01) and rural older adults ( β = 0.379, *p* < 0.01). Basic older adult care services have a significant positive effect on the quality of life of rural older adults ( β = 0.168, *p* < 0.01), but not on urban older adults ( β = − 0.017, *p* > 0.1). However, extended older adult care services have a greater role in improving the quality of life of rural older adults ( β = 0.387, *p* < 0.01) and urban older adults ( β = 0.229, *p* < 0.01).

#### 4.4.3 Disablement status group differences

Exploring the influence of community older adult care services on the quality of life of the disablement status group helps provide more high-quality and efficient older adult care services. According to the question “Do you need help in your daily life?.” If the respondent answered “yes,” it was considered “disabled,” and if the respondent answered “no,” it was considered “non-disabled.” In the group of non-disabled older adults, total community older adult care services ( β = 0.295, *p* < 0.01), basic community older adult care services ( β = 0.065, *p* < 0.05), and extended community older adult care services ( β = 0.309, *p* < 0.01) significantly improve their quality of life. In the group of disabled older adults, the coefficient of total community older adult care services and extended community older adult care services on quality of life is 0.219 and 0.193, which are significant at the 5% level. However, there is no significant correlation between basic older adult care services and their quality of life ( β = − 0.118, *p* > 0.01), which indicates that basic community older adult care services do not have a significant effect on the quality of life of disabled older adults.

### 4.5 Mechanism test

**Figure 1:**
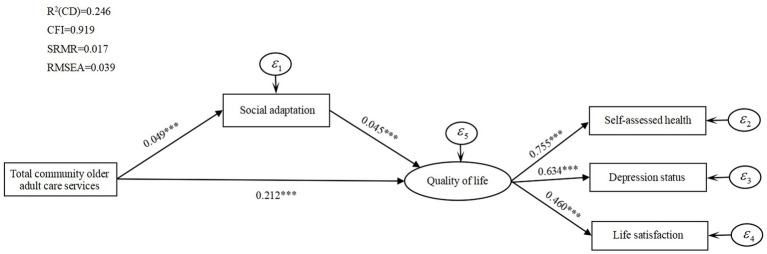
Total community older adult care services, social adaptation, and quality of life mediated effects path analysis.

**Figure 2:**
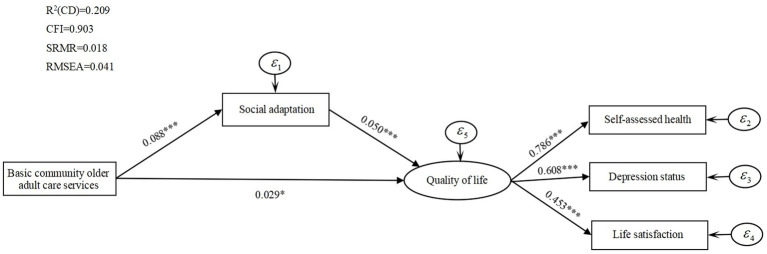
Basic community older adult care services, social adaptation, and quality of life mediated effects path analysis.

**Figure 3:**
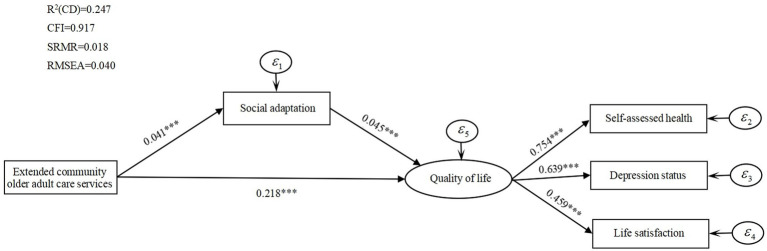
Extended community older adult care services, social adaptation, and quality of life mediated effects path analysis.

To further test the intrinsic influence mechanism of the three types of community older adult care services on older adults’ quality of life, we add the mediating variable social adaptation and test its mediating effect. To provide a more intuitive understanding of the mediating effect of social adaptation, Figures 1–3 report the standardized coefficients of the structural equation model incorporating the mediating variable social adaptation. Control variables are included in the model, but they are not presented in these figures to show the main path of analysis more clearly.

Figure 1 shows that the direct influence coefficient of total community older adult care services on the quality of life of older adults is 0.212, which is significant at 1% level. The influence coefficient of total community older adult care services on social adaptation is significantly positive at 1% ( β = 0.049, *p* < 0.01). At the same time, social adaptation has a significant impact on quality of life ( β = 0.045, *p* < 0.01). Thus, the results suggest that social adaptation is the mediating mechanism that affects community older adult care services and the quality of life of older adults. In Figure 2, the basic older adult care services significantly positively correlated with quality of life ( β = 0.029, *p* < 0.1) in the direct influence path. In the indirect influence path, the influence coefficient of basic older adult care services on social adaptation is 0.088, which is significant at 1% level, and the influence coefficient of social adaptation on quality of life is 0.050, which is significant at 1% level. It can be seen that social adaptation plays a mediating role in basic community older adult care services and the quality of life of older adults. Figure 3 shows that the direct influence coefficient of extended community older adult care services on the quality of life is 0.218, which is significant at 1% level. Extended older adult care services affect older adults’ social adaptation at the 1% significance level ( β = 0.041, *p* < 0.01), and social adaptation affects quality of life at the 1% significance level ( β = 0.045, *p* < 0.01). These results suggest that social adaptation plays a mediating role in extended community older adult care services and the quality of life of older adults.

All the above results validate the mediating role of social adaptation between three types of community older adult care services and the quality of life of older adults.

Table 7 reports the mediating effects of social adaptation on the quality of life of older adults. By analyzing the mediating effects, the mediating effects of three community older adult care services on quality of life do not contain 0 at the 95% confidence interval. The paths of community older adult care services on quality of life reach the level of significance, which suggests that there is a partially mediating effect of social adaptation between the effects of three types of community older adult care services on quality of life.

**Table 7:** Direct and indirect effects and Intermediation effect interval for the modal.

Variable	Direct effect	Indirect effect	Total effect	Intermediation effect interval
Total community older adult care services	0.288*** (0.021)	0.003*** (0.001)	0.291*** (0.021)	[0.000,0.006]
Basic community older adult care services	0.048* (0.027)	0.007*** (0.003)	0.056** (0.027)	[0.002,0.012]
Extended community older adult care services	0.300*** (0.021)	0.003*** (0.001)	0.302*** (0.021)	[0.000,0.005]
*N*	4,652			

*p < 0.1, **p < 0.05, ***p < 0.01. Numbers in parentheses are robustness standard errors.

## 5 Discussion

Through empirical analysis of CLASS2020 data, this study explores the linkage between community older adult care services and the quality of life of older adults, as well as older adults’ social adaptation, paying special attention to its mechanism. We draw the following conclusions.

First of all, community older adult care services are positively correlated with the quality of life of older adults, which is consistent with the previous findings (43). They indicate that community older adult care services enable older adults to enjoy convenient services in a familiar environment, which is beneficial to improving their quality of life (44). It also improves older adults’ living conditions, allows them to have a better sense of experience, increases their sense of security in life, and thus elevates their quality of life (29). In addition to this, we also found that different types of community older adult care services have different impacts on the quality of life of older adults. Extended older adult care services have a more significant positive effect on quality of life compared to basic older adult care services. As they grow older, older adults are easily satisfied with their material life, but they are more likely to encounter social isolation dilemmas such as a lack of interpersonal communication and increased loneliness (45). In modern society, the needs of older adults for a spiritual life are constantly rising, and they are eager to get more care, companionship, and respect. On the one hand, with the improvement of living standards and medical conditions, their life expectancy has been prolonged (46), but due to the decline of physical functions and the change of social roles, older adults are more prone to loneliness, anxiety and other psychological problems (47). On the other hand, the changes in the structure of the modern family, such as the reduction in the number of children, the downsizing of the family, and the children’s busy work schedule, make older adults less emotionally supportive of the family (48). Therefore, the provision of extended older adult care services by the community can satisfy their emotional need, provide them with the necessary psychological care and companionship, alleviate their loneliness and depressing emotions, and improve their quality of life.

Secondly, our study suggests that there is heterogeneity in the impact of the three types of community older adult care services on the quality of life of older adults. Within the subgroup of age, total and extended older adult care services have a significant impact on the quality of life of younger old adults and older to oldest old adults. However, basic older adult care services do not have a significant effect on the quality of life of older to oldest old adults. This may be because younger older adults have relatively better physical functions and self-care abilities than their senior counterparts, and are more capable of adapting to the various contents and forms of community older adult care services. Within the subgroup of registered residents, the results show that the three types of community older adult care services can improve the quality of life of the rural older adults. For the urban older adults, the effect of total and extended community older adult care services on their quality of life is more significant. By contrast, basic older adult care services do not have a significant effect on the quality of life of the urban older adults. This is because of the low level of public services, weak infrastructure, inconvenient living in rural areas of China, as well as the lack of care for older adults due to their children working outside the home all year round (49), which leads to greater need of services for the rural older adults. As the basic material needs of urban older adults have been fundamentally met, it is easier for them to develop higher-level psychological needs (50), and extended older adult care services can better meet the needs of the urban older adults. Within the subgroup of disablement status, for non-disabled older adults, three types of community older adult care services can significantly improve their quality of life. Whereas, for the disabled older adults, basic older adult care services have no significant impact on their quality of life, but the extended older adult care services are positively correlated with quality of life. This may be because the disabled older adults have impaired self-care functions, and they need long-term and professional care, however, the simple basic community older adult care service for the aged makes it difficult to meet their needs (51). Moreover, their activities are restricted resulting in little communication with the outside world (52). As a result, they tend to have negative emotions such as depression and loneliness compared to other older adults. Thus, they have less demand for basic older adult care services, but more demand for extended older adult care services.

Finally, social adaptation plays a mediating role in the three types of community older adult care services and the quality of life of older adults. The results of this study suggest that community older adult care services help older adults better integrate into society, and thus improve their quality of life. Community older adult care services have a positive impact on the social adaptation of older adults. On the one hand, the community provides convenient living services for older adults, making it easier for them to access the resources they need in their daily lives, enabling them to face the various challenges and changes in their lives with greater confidence, and helping them to better adapt to society (53); On the other hand, the provision of community older adult care services also reflects society’s concern about older adults, giving social support to them, improving older people’s perception of society, enhancing their sense of belonging to society and increasing trust in society, which is conducive to their integration into society (54). Thus, they can reduce their doubts and uneasiness to society and enhance their ability to adapt to society. Older adults with strong social adaptability can better adapt to the social environment and actively participate in social activities, which will help them to increase physical activity, improve physical fitness, reduce negative emotions, and elevate their sense of happiness. Consequently, the overall quality of life is improved.

However, there are some limitations in this study. Firstly, this is a cross-sectional study. It is difficult to observe the dynamic changes of community older adult care services on the quality of life of older adults, and the use of panel data can better improve the robustness of the results. Secondly, due to the limitations of the public survey data, there are some defects in the selection of variables for the study, and it is difficult to comprehensively reflect the real situation of the quality of life of older adults. In the future should be changed to use more rigorous scientific measurement tools. Thirdly, the data are obtained from China, the national conditions of each country are different, and the direct generalization of these conclusions to other cultural environments may face a number of challenges. To ensure the generalizability and validity of the findings, we need to conduct further in-depth research to comprehensively test the applicability of these findings in different socio-cultural contexts. Finally, this paper focuses on quantitative analysis by constructing structural equation modeling, and there are still some limitations. Future research can further integrate qualitative research methods. Through data triangulation and cross-validation of results, an in-depth understanding of the underlying structure as well as the complex relationships among variables can be achieved. This integration helps to break through the limitations of a single methodology and analyze the research problem from multiple perspectives, thus developing a more comprehensive and in-depth understanding of the relevant phenomena and mechanisms.

## 6 Conclusion

In summary, it is found that three types of community older adult care services can directly and positively affect the quality of life of older adults, in which extended older adult care services have a more significant effect on the quality of life than basic older adult care services. The effects of three types of community older adult care services on quality of life are heterogeneous depending on age, registered residence and disablement status. Additionally, social adaptation plays a mediator role between the three types of community older adult care services and the quality of life of older adults. Through the provision of comprehensive older adult care services, the community helps older adults to better adapt to the social environment, thereby enhancing their standard of living and improving their quality of life.

Therefore, the government should consider the following three aspects when formulating policies. Firstly, increase the effective supply of extended community older adult care services. Through the above research, it is found that extended community older adult care services have the most obvious effect on the improvement of the quality of life of the older adults. Thus, it is necessary to increase the supply of extended community older adult care services. On the one hand, the government provides policies and financial support for extended community older adult care services. On the other hand, extensive integration of social resources. Through government purchase of services, community volunteer services, market operation and other diversified ways, to expand the coverage of extended services, enrich the content of extended services, and promote the development of extended older adult care services. Then, differentiated and precise supply should be implemented. In view of the heterogeneity of the impact of community older adult care services on quality of life among different groups of older persons, differentiated and precise strategies must be adopted in the delivery of services. Community older adult care services should be rationally allocated according to the diverse needs of older adults, so as to better meet the personalized requirements of different senior groups. Finally, improve the quality of community aged care service. We found that community older adult care services have a positive impact on the quality of life of the older adults, and social adaptation plays a key mediating role in this positive impact process. By strengthening the professional training of service personnel, introducing scientific and technological means, and reinforcing the supervision of services, the quality of older adult care services can be continuously improved. High-quality older adult care services bring better service experience to the older adults, elevate their satisfaction levels and improve their impression on society, so as to help the older adults better integrate into and adapt to the social environment, and effectively improve the quality of life of the older adults by promoting their social adaptation.

## Data Availability

The original contributions presented in the study are included in the article/supplementary material, further inquiries can be directed to the corresponding author.
